# Assessment of the Hypoglycemic and Hypolipidemic Activity of Flavonoid-Rich Extract from *Angelica keiskei*

**DOI:** 10.3390/molecules27196625

**Published:** 2022-10-06

**Authors:** Lanlan Tu, Rui Wang, Zheng Fang, Mengge Sun, Xiaohui Sun, Jinhong Wu, Yali Dang, Jianhua Liu

**Affiliations:** 1 Department of Food Science and Engineering, School of Agriculture and Biology, Shanghai Jiao Tong University, Shanghai 200240, China; 2 State Key Laboratory for Managing Biotic and Chemical Threats to the Quality and Safety of Agro-Products, College of Food and Pharmaceutical Sciences, Ningbo University, Ningbo 315211, China; 3 Department of Laboratory Medicine, School of Medicine, Institute of Molecular Medicine, Shanghai Jiao Tong University, Shanghai 200240, China; 4 International Faculty of Applied Technology, Yibin University, Yibin 644000, China

**Keywords:** hypoglycemic activity, hypolipidemic activity, *Angelica keiskei*, flavonoid, function evaluation

## Abstract

*Angelica keiskei* contains a variety of bioactive compounds including chalcone, coumarin, and phytochemicals, endowing it with pharmacological effects such as lipid-lowering activity, antitumor activity, liver protection, and nerve protection. This study aims to study the hypoglycemic and hypolipidemic effects of the flavonoid-rich extract from *Angelica keiskei* (FEAK) in an effort to exploit new applications of FEAK and increase its commercial value. In this paper, flavonoid compounds in *Angelica keiskei* were extracted using 50% ethanol, and the contents of the flavonoid compounds were analyzed by UPLC-MS/MS. Then, the hypoglycemic and hypolipidemic activities of the FEAK were investigated through in vitro enzyme activity and cell experiments as well as establishing in vivo zebrafish and Caenorhabditis elegans (*C. elegans*) models. The UPLC-MS/MS results show that the major flavonoid compounds in the FEAK were aureusidin, xanthoangelol, kaempferol, luteolin, and quercetin. The inhibitory rates of the FEAK on the activity of α-amylase and cholesterol esterase were 57.13% and 72.11%, respectively. In cell lipid-lowering experiments, the FEAK significantly reduced the total cholesterol (TC) and total triglyceride (TG) levels in a dose-dependent manner, with 150 μg/mL of FEAK decreasing the intracellular levels of TC and TG by 33.86% and 27.89%, respectively. The fluorescence intensity of the FEAK group was 68.12% higher than that of the control group, indicating that the FEAK exhibited hypoglycemic effects. When the concentration of the FEAK reached 500 μg/mL, the hypoglycemic effect on zebrafish reached up to 57.7%, and the average fluorescence intensity of *C. elegans* in the FEAK group was 17% lower than that of the control group. The results indicate that the FEAK had hypoglycemic and hypolipidemic activities. The findings of this study provide theoretical references for the high-value utilization of *Angelica keiskei* and the development of natural functional food with hypoglycemic and hypolipidemic activities.

## 1. Introduction

Diabetes is a metabolic disease characterized by abnormal glucose metabolism, and type 2 diabetes accounts for about 90% of all diabetes patients [1]. Insulin resistance, one of the most prominent features of type 2 diabetes, mainly results from disorders of lipid metabolism, which is, in turn, one of the frequent complications of diabetes. According to a China Health and Nutrition Survey, a total of 20~90% of diabetes patients suffer from hyperlipidemia [2]. Therefore, the prevention and treatment of type 2 diabetes as well as lipid metabolism disorders are of utmost importance. At present, metformin and acarbose tablets are proven to be relatively reliably safe long-term in prediabetic patients, while there is insufficient evidence for the long-term safety of other drugs [3]. The drugs used to regulate blood lipids, including statins and bast, may cause adverse reactions such as liver injury and rhabdomyolysis, despite their high efficacy [4]. It has been reported that traditional Chinese medicine treatment is a useful therapeutic option for regulating and treating type 2 diabetes patients with hyperlipidemia because of its safety and efficacy [5]. In addition to conventional drug therapy, functional foods have become increasingly attractive options for helping lower blood sugar and fat.

*Angelica keiskei* is a perennial herb plant from the Umbelliferae family that can be used as both medicine and food [6]. Praised in “Foods for Health in the 21st century” [7], *Angelica keiskei* has pharmacological functions such as antitumor and anti-inflammatory activities, and it can lower hypertension, hyperlipidemia, and hyperglycemia, prevent cardiovascular and cerebrovascular diseases, regulate the intestinal tract, and improve human immunity and sleep quality.

*Angelica keiskei* is a rich source of flavonoid compounds. Studies have shown that flavonoid compounds possess a wide range of pharmacological activities, such as vasodilation, lipid-lowering activity, anticoagulation, anti-inflammation, antitumor activity, analgesia, and nonenzymatic glycosylation, and these properties have become one of the focuses of phytochemical studies. Riezki Amalia et al. showed that extract from *Angelica keiskei* stems protects HEK293 cells from N-acetyl-*p*-benzoquinone imine (NAPQI) damage and has nephroprotective properties [8]. Zhang studied the effect of chalcone from *Angelica keiskei* on the lipid metabolism in rats with type 2 diabetes. Compared with the diabetic model group, the serum levels of the TG, TC and FFA contents were significantly lower in the intervention groups [9]. Zhang found that a flavonoid-rich ethanol extract from *Angelica keiskei* leaves in a dose of 800 mg/kg could exhibit the same effect on TC with metformin [10]. Liu studied the effect of chalcones extracted from *Angelica keiskei* (AC) on the hepatocytes of rats with type 2 diabetes. Compared with rats in the diabetic control group, the levels of blood glucose and serum insulin in the 10 mg/kg AC group were decreased [11].

Most of the reports were about the single functional study of *Angelica keiskei*, and the simultaneous glucose-lowering and lipid-lowering effects of the *Angelica keiskei* extract were not investigated. However, further development of *Angelica keiskei* as a natural functional food requires systematic and comprehensive studies of its hypoglycemic and hypolipidemic activities, including its detailed flavonoid composition and the dose–effect relationship of the hypoglycemic and hypolipidemic activities of the flavonoid-rich extract. Therefore, this study aimed to assess the hypoglycemic and hypolipidemic effects of the flavonoid extract obtained from *Angelica keiskei* by in vitro and in vivo experiments so as to lay the foundation for further development of the functional food or drugs from *Angelica keiskei*.

## 2. Results and Discussions

### 2.1. The Flavonoid Compositions of FEAK

UPLC-MS/MS was used to analyze the components of flavonoids in the FEAK. As can be seen from Table 1, the most abundant flavonoids were aureusidin, xanthoangelol, kaempferol, luteolin, and quercetin. Studies have shown that aureusidin, xanthoangelol, and quercetin play a crucial role in regulating the cardiovascular system through the inhibition of blood lipids, prevention of the elevation of low-density lipoprotein in serum, and reduction in blood sugar and serum cholesterol. Wang Junbo [10] showed that 10 μg/mL luteolin and quercetin cholesterol significantly reduce the TG content in HepG2 cells. Busu et al. [12] showed that quercetin and kaempferol significantly inhibit the expression of C/EBPα, PPARγ, and SPEBP-1c at high concentrations, thus improving lipid metabolism and preventing lipid overaccumulation. The flavonoid composition of FEAK, therefore, supports its potential application in the development of functional material with hypoglycemic and lipid-lowering activities.

### 2.2. The Inhibition Activities on the α-Amylase and Cholesterol Esterase

**Figure 1:**
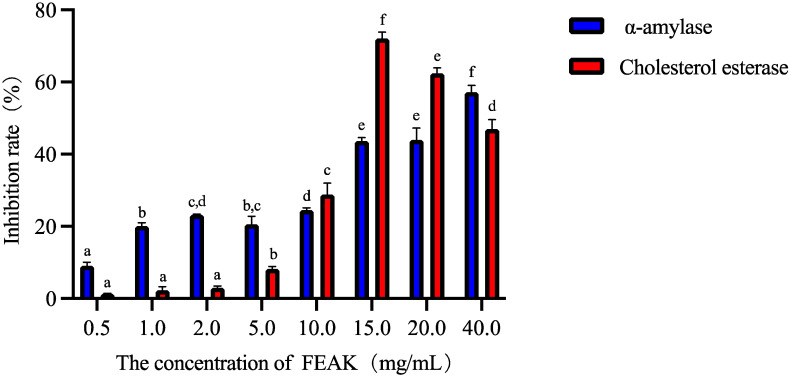
The inhibitory rate of the FEAK on the activity of α-amylase and cholesterol esterase. Note: different lowercase letters in the same enzyme activity test group represent the existence of significant differences at *p* < 0.05 in the different concentration group of the FEAK.

α-amylase inhibitors destroy the α-1,4-glycosidic bond of starch in the intestinal tract and are considered as therapeutic targets for type 2 diabetes [13]. Therefore, the inhibition of α-amylase activity is of great significance for the study of hypoglycemia. Figure 1 shows that, with increasing concentrations of the FEAK, the inhibitory rate of the FEAK on α-amylase significantly increased overall. When the concentration was 40 mg/mL, the inhibition rate of α-amylase reached 57.13 ± 1.88%.

Moreover, pancreatic cholesterol esterase contributes to the bile salt-dependent hydrolysis of dietary cholesterol esters as well as the hydrolysis of triglyceride-phospholipids which are potential targets for the prevention of dietary cholesterol absorption [14]. Figure 1 shows that the inhibitory rate of the FEAK on the activity of cholesterol esterase first increased and then decreased with the increasing concentration. When the concentration of the FEAK was increased to 15 mg/mL, the inhibition rate reached a maximum value of 72.11 ± 1.69%. These results demonstrate that FEAK acts as both an α-amylase inhibitor and a cholesterol esterase inhibitor.

### 2.3. Effect of FEAK on the Intracellular Levels of TC and TG in HepG2 Cells

Blood fat is the general name for various lipid substances in blood, among which TC and TG are the most important and abundant, so they are important routine clinical test indices of blood lipid levels [15]. There have been many studies on the hypolipidemic activity of flavonoids. The TG and FFA levels in cells and FFA levels in the cell supernatant in cells treated with low, medium, and high doses of total chamomile flavonoids are significantly reduced [16]. Kobayashi et al. found that persimmon flavone regulates the expression of cholesterol 7α strengthening the enzyme gene and the cholesterol regulatory primary binding protein (SREBP) gene to affect the lipoprotein receptor [17]. Liu Chang showed that 200 μg/mL of mangrove berry extract reduces lipid and TG levels in HepG2 cells by 25.93% and 37.23%, respectively [18]. Lv Yichun et al. showed that 100 μg/mL of blueberry polyphenols decrease TG levels in liver cells by 40%, demonstrating that these compounds can clear fat accumulation in liver cells [19].

**Figure 2:**
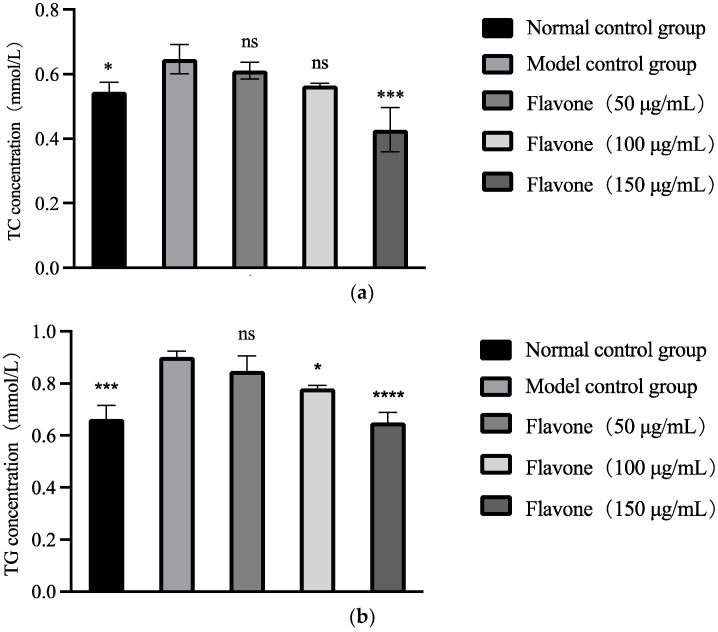
Changes of TC (**a**) and TG (**b**) values in HepG2 cells after treatment of FEAK. Compared with the model control groups, * *p* < 0.05, *** *p* < 0.001, **** *p* < 0.0001.

Figure 2 shows the effect of the FEAK on the TC and TG levels in the HepG2 cells. Compared with the normal control groups, the TC and TG levels in the sodium oleate and sodium palmitate model groups were significantly increased (*p* < 0.05), while the levels in the groups treated with the FEAK were significantly decreased. Compared with the model control groups, the change in TC and TG levels showed a dose-dependent relationship. When the concentration of the FEAK increased to 150 μg/mL, the intracellular levels of TC in the HepG2 cells decreased by 33.86% (*p* < 0.001). When the concentration of the FEAK increased to 150 μg/mL, the intracellular levels of TG in the HepG2 cells decreased by 27.89% (*p* < 0.001).

### 2.4. Effect of FEAK on Glucose Uptake in HepG2 Cells

In a glucose uptake model using HepG2 cells, hypoglycemic ingredients were added, the cells in each group were treated with fluorescent-labeled glucose, and the fluorescence intensity of the cells was measured using flow cytometry. The fluorescence intensity of the treated cells was higher than that of the control group, indicating that the cells treated with hypoglycemic ingredients absorbed more glucose. These observations reflect the increase in the glucose utilization rate of cells in the human body and the subsequent decrease in blood glucose, demonstrating that the samples were exhibiting hypoglycemic effects [20].

According to Table 2, increasing the FEAK leads to stronger fluorescence intensity, and the change due to the FEAK treatment was higher than that of the contrast group. When the concentration of the FEAK reached 100 mg/mL, the contrast value reached the highest value of 68.12% (*p* < 0.01). This result indicates that the FEAK absorbs the glucose in cells and exerts a hypoglycemic effect.

### 2.5. Evaluation of Hypoglycemic Efficacy of FEAK In Vivo in a Zebrafish Model

Zebrafish are often used as a hypoglycemic model for the research of human diabetes drugs because they employ similar mechanisms of blood glucose regulation to mammals [21]. Zebrafish are an ideal model with which to study diabetes because of their high carbohydrate diet. Sugar in food is metabolized under catalytic enzymes, such as hexokinase and glucose 6 phosphatases, via glycometabolism. Therefore, the lack of any intermediate steps in the carbohydrate metabolism will have irreversible effects on the nervous system and the development of zebrafish.

Several zebrafish models of diabetes have been established. Gleeson et al. [22] induced type 2 diabetes in zebrafish by soaking the fish in a 2% glucose solution, but the induction period of this method is long (28 days). Capiotti et al. [23] established a hyperglycemia model of zebrafish by soaking the fish in a 0.111 mol/L glucose solution for 14 days. This induction cycle is relatively short, but the model is unstable, and the glucose concentration is high, which can lead to the death of the zebrafish. In this study, a zebrafish hyperglycemia model was established by combining glucose solution immersion and egg yolk powder feeding. The hypoglycemic effect of 125~500 μg/mL of FEAK in vivo was evaluated with this model.

**Figure 3:**
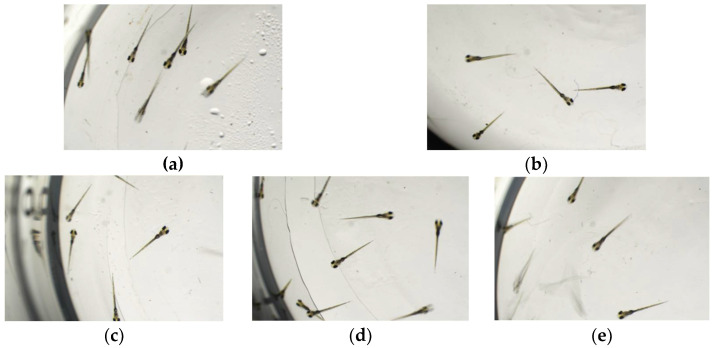

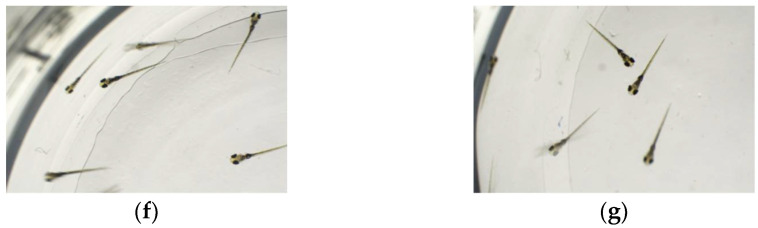
The phenotypic appearance of zebrafish fed with different concentration of FEAK. (**a**) Normal control group. (**b**) Model control group. (**c**) 31.2 μg/mL of FEAK group. (**d**) 62.5 μg/mL of FEAK group. (**e**) 125 μg/mL of FEAK group. (**f**) 250 μg/mL of FEAK group. (**g**) 500 μg/mL of FEAK group.

To determine the maximum tolerance concentration (MTC) of the FEAK in the zebrafish model experiment, the mortality rate of adult zebrafish was calculated 2 days after the FEAK administration (Table 3). No death was found in the concentration range from 31.2 µg/mL to 500 µg/mL, and the phenotypic appearance, feeding and swimming abilities of the treated fish were the same as those of the control group (Figure 3), indicating that the FEAK is not toxic to adult zebrafish. As 500 µg/mL is a large dose, the MTC of the FEAK was considered to be 500 µg/mL and was used to determine the experimental doses.

As shown in Table 4, the blood glucose level of zebrafish in the hyperglycemia group was 2.31 ± 0.129 mmol/L, which is about 2.5 times that of zebrafish in the normal control group (0.920 ± 0.044 mmol/L, *p* < 0.001), demonstrating that the hyperglycemia model was successful. As a short-term measurement of blood glucose, blood glucose levels can be directly used for blood glucose monitoring. Blood glucose levels were measured after zebrafish in the hyperglycemia group were treated with 125, 250, or 500 μg/mL of the FEAK for 2 days. Our results show that the hypoglycemic effect improved with increasing concentrations of the FEAK, indicating that the hypoglycemic effect of FEAK is dose-dependent. The blood glucose levels of the zebrafish dropped by 57.7% upon treatment with 500 μg/mL of FEAK (*p* < 0.001), and this hypoglycemic effect was similar to that of the positive control drug pioglitazone hydrochloride.

### 2.6. Evaluation of Hypolipidemic Efficacy of FEAK In Vivo in a C. elegans Model

**Figure 4:**
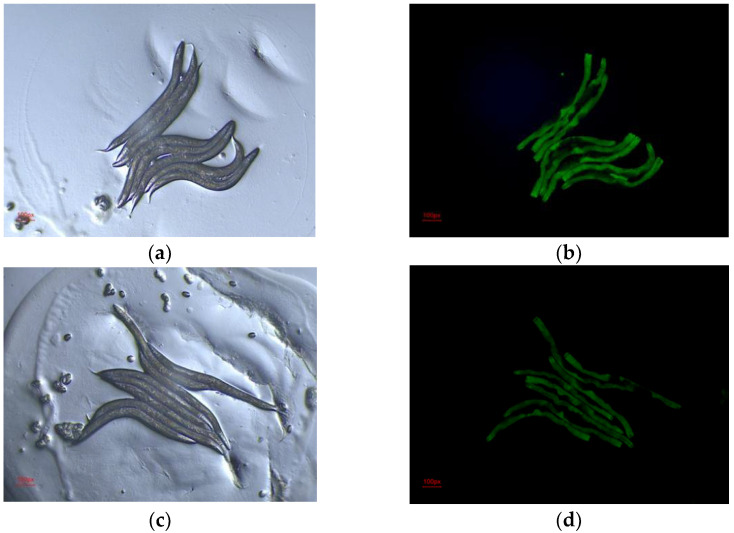
Representative images of dhs-3::gfp fluorescence in worms induced by DMSO and FEAK. (**a**) DMSO group (bright field, 100 px). (**b**) DMSO group (dark field,100 px). (**c**) FEAK group (bright field). (**d**) FEAK group (dark field).

*C. elegans* is a good model organism for studies of lipid storage because it possesses similar regulatory factors and metabolic pathways that regulate adipose deposition and related metabolic diseases to mammals. Many mutants of lipid deposition have been generated in *C. elegans.* The dhs-3::gfp mutant exhibits green fluorescence on the surface of lipid droplets, allowing changes in lipid deposition in *C. elegans* to be easily observed. Therefore, we used dhs-3::gfp mutants to observe the effect of the FEAK on lipid accumulation in *C. elegans*. The fluorescence images were processed using ImageJ. The results in Figure 4 show that the mean fluorescence intensity of the DMSO group was 15.10 ± 0.086, while the mean fluorescence intensity of the 500 μg/mL FEAK group was 12.56 ± 0.108 (*p* < 0.001), a reduction of almost 17%. This result indicates that the FEAK decreases lipid accumulation in *C. elegans* and suggests that the FEAK lowers adipose deposition in vivo.

## 3. Materials and Methods

### 3.1. Materials and Chemicals

*Angelicakeiskei* (Xiancao Health Management Group Co., Ltd., Shandong, China); human hepatoma cells (HepG2) (Yongchuan Biotechnology, Shanghai, China); sodium oleate (Sigma, St. Louis, MO, USA); sodium palmitate (Sigma, St. Louis, MO, USA)); Dulbecco’s modified Eagle medium (DMEM) (Hyclone, Beijing, China); TC and TG assay kits (Nanjing Jiancheng Biology Co., Ltd. Nanjing, China); α-Amylase (Yuanye Biology Co., Ltd. Shanghai, China); cholesterol esterase (Yuanye biology Co., Ltd. Shanghai, China); zebrafish (AB wild-type, Shanghai Feixi Biotechnology Co., Ltd. Shanghai, China); Caenorhabditis elegans (presented by the Wu Ziyun research group of Shanghai Jiaotong University, Shanghai, China); M9 buffer: Na_2_HPO_4_·12H_2_O 0.15 g, KH_2_PO_4_ 0.03 g, NaCl 0.05 g, MgSO_4_ 0.0025 g; and pure water to a final volume of 100 mL.

### 3.2. Preparation of Flavonoid-Rich Extract from Angelica keiskei

*Angelica keiskei* samples were shattered after vacuum freeze-drying for 24 h. The dried samples were milled to a powder using a 100-mesh sieve. The powder was mixed with 50% ethanol at a mass/volume ratio of 1:10 (g:mL) and was heated and stirred in a water bath at 37 °C for 3 h. After cooling to room temperature, the extraction was collected by centrifuging at 8000 rpm/min at 4 °C. The ethanol extract process was repeated, and all extracts were placed in a rotary evaporator to remove trace amounts of ethanol. After lyophilization, the flavonoid-rich extract from *Angelica keiskei* (FEAK) was obtained and stored at −20 °C [24].

### 3.3. Analysis of the Flavonoid Composition of FEAK

FEAK samples were ground to a powder in a laboratory mill (30 Hz, 1.5 min). Then, 100 mg of powder was weighed and dissolved in 1.2 mL 70% methanol, stirred every 30 min for 30 s 6 times. The extract sample was placed in a refrigerator at 4 °C overnight. After centrifugation (12,000 rpm, 10 min), the supernatant was obtained, and the sample was filtered through a 0.22 μm microporous membrane and stored in the sample bottle for UPLC-MS/MS [25].

The UPLC-MS/MS analytical conditions were as follows. The UPLC was equipped with an Agilent SB-C18 column (1.8 µm, 2.1 mm × 100 mm). The mobile phase consisted of solvent A, pure water with 0.1% formic acid, and solvent B, acetonitrile with 0.1% formic acid. Sample measurements were performed with a gradient program that employed the starting conditions of 95% A, 5% B. Within 9 min, a linear gradient to 5% A, 95% B was programmed, and a composition of 5% A, 95% B was kept for 1 min. Subsequently, 95% A, 5.0% B was reached within 1.1 min and kept for 2.9 min. The flow velocity was set to 0.35 mL per minute. The column oven was set to 40 °C. The injection volume was 2 μL. The effluent was alternatively connected to an ESI-triple quadrupole-linear ion trap (QTRAP)-MS.

The relative content of flavonoid compound above 1% in the extract was detected by a relative quantification method, namely, it was calculated by the ratio of the peak area of each flavonoid component divided by the peak areas of all flavonoid components.

### 3.4. Evaluation of the Hypoglycemic and Hypolipidemic Activity of FEAK In Vitro

#### 3.4.1. Measurement of α-Amylase Inhibitory Activity

We used the method of Yuca et al. [26], with slight modifications. The α-amylase solution (1 U/mL) was mixed into samples at varying concentrations and preheated in 37 °C water bath for 5 min, followed by addition of 1% preheated starch solution. The solution was incubated in a 37 °C water bath for 5 min. Then, 0.25 mL DNS solution was immediately added for color responses, and the reaction mixtures were incubated in boiling water for 5 min. After cooling in an ice bath, the reaction mixtures were diluted to 5 mL with distilled water. The absorbance was recorded at 540 nm on a microplate reader. The enzyme solution and starch solution were prepared in PBS buffer at pH 6.8, 0.1 mol/L. The additive and concentration of each tube are shown in Table 5. Equation (1) was used to calculate percent inhibition.

(1) $$\mathrm{Inhibitory}\;\mathrm{activity}=1-\frac{A_{3}-A_{4}}{A_{1}-A_{2}}\times 100\%$$

A_1_ is the absorbance of the blank group (equal volume buffer replaces the sample solution); A_2_ is the absorbance of blank control group (equal volume buffer replaces sample solution and enzyme solution); A_3_ is the absorbance of the sample group; and A_4_ is the absorbance of the sample control group (equal volume buffer instead of enzyme solution).

#### 3.4.2. Measurement of Cholesterol Esterase Inhibitory Activity in Porcine Pancreas

We used the method of Su et al. [27], with slight modifications. In brief, the measurement was performed in 0.1 mol/L sodium phosphate (pH 7.04) containing 0.1 mol/L NaCl, 0.2 mmol/L *p*-nitrophenyl butyrate (*p*-NPB) and 5.16 mmol/L sodium taurocholate buffer (STC). First, 10 U/mL of porcine pancreatic cholesterol esterase and PNPB were predissolved in acetonitrile and stored at −20 °C. Porcine pancreatic cholesterol esterase was added to the reaction tube, and samples were incubated at 25 °C for 5 min. Then, the absorbance of the solutions was measured at 450 nm using an ultraviolet-visible spectrophotometer. The additives and corresponding concentrations of each tube are shown in Table 6. Equation (1) was used to calculate percent inhibition.

#### 3.4.3. Glucose Consumption Assay in HepG-2 Cells

The effect of FEAK on glucose consumption was investigated in insulin-resistant HepG-2 cells. After thawing, subculturing, and plating, HepG-2 cells were processed as follows. First, 100 nmol/L of recombinant human insulin was added, and cells were incubated at 37 °C for 30 min. Then, 0, 10, 25, 50 to 100 mg/mL FEAK was added, and cells were incubated at 37 °C for 1 h. Finally, 50 μM 2-NbDG was added, and cells were incubated at 37 °C for 1 h. After the incubation, the culture medium was removed, and cells were washed with PBS buffer twice. After digestion with 1 mL trypsin-EDTA solution, culture medium was added, and the resulting single cell suspension was placed in a centrifugal tube and centrifuged at 1000 RPM for 5 min. Afterward, the supernatant was removed and discarded, and the solution was centrifuged at 1000 rpm for 5 min. This process was repeated three times. HepG-2 cells were collected into flow cytometry tubes and placed on ice to maintain a low temperature for further determination of cell fluorescence intensity [28].

Fluorescence intensity was measured by flow cytometry in specific channels (10,000 cells per tube). The contrast value of fluorescence intensity was calculated according to the following Equation (2):

(2) $$\delta =\frac{\;\mathrm{Fluorescence}\;\mathrm{intensity}\;\mathrm{of}\;\mathrm{samples}\;-\;\mathrm{Fluorescence}\;\mathrm{intensity}\;\mathrm{of}\;\mathrm{control}\;\mathrm{group}}{\mathrm{Fluorescence}\;\mathrm{intensity}\;\mathrm{of}\;\mathrm{control}\;\mathrm{group}}\times 100\%$$

The larger the value of δ, the stronger the ability of cells to absorb glucose and the stronger the hypoglycemic effect [20].

#### 3.4.4. Total Cholesterol (TC) and Triglyceride (TG) Assay in HepG-2 Cells

The high lipid cell culture medium consisted of DMEM supplemented with 500 μmol/L sodium oleate and 250 μmol/L sodium palmitate [7]. HepG-2 cells were treated with 0.25% trypsin and transferred to petri dishes. The sample dose was set based on the cytotoxicity test. The blank control group and the model group were cultured in basal DMEM medium and high lipid medium at 37 °C and 5% CO_2_ for 24 h, respectively. The sample intervention groups were first cultured in high lipid medium at 37 °C and 5% CO_2_ for 24 h, then 50, 100, or 150 μg/mL FEAK was added for an additional 24 h [12].

After HepG-2 cells were cultured, the culture medium was discarded. The cells were washed with the PBS at 4 °C 3 times. Radio immunoprecipitation assay (RIPA)lysis buffer was added to each petri dish, and the cells were placed on ice for 30 min. The lysate was collected and centrifuged at 10,000 rpm for 10 min at 4 °C. The supernatant was obtained for the determination of TG and TC levels by using TC and TG assay kits.

### 3.5. Experimental Analysis of In Vivo Hypoglycemic Effect in a Zebrafish Model

#### 3.5.1. Sample Preparation

Sample group: 50.0 mg/mL mother liquor was prepared using ethanol extract of FEAK and DMSO and stored at −20 °C.

Positive control group: 10.0 mg/mL mother liquor was prepared using pioglitazone hydrochloride tablets and DMSO and stored at −20 °C.

#### 3.5.2. Evaluation of the Hypoglycemic Effect of FEAK in a Diabetic Zebrafish Model

Zebrafish of the AB wild-type strain were bred naturally in pairs. Zebrafish were all raised in fish culture water at 28 °C. Zebrafish at 5 days postfertilization (5 DPF) were used to evaluate the maximum tolerable concentration (MTC) and auxiliary hypoglycemic efficacy of FEAK.

The MTC of FEAK was determined. Zebrafish of the AB wild-type strain at 5 DPF were randomly selected and placed in 25 mL beakers, with 30 zebrafish in each beaker (experimental group). FEAK was dissolved in the water (31.2, 62.5, 125, 250, and 500 μg/mL). All experimental groups except for the normal control group were given 0.15% yolk powder solution in the daytime, and 3% glucose solution was given in the evening after the removal of the yolk powder solution to establish the zebrafish hyperglycemia model. After treatment at 28 °C for 48 h, the MTC of FEAK on zebrafish in the model control group was determined.

The auxiliary hypoglycemic efficacy of FEAK was further evaluated. Zebrafish of the AB wild-type strain at 5 DPF were randomly selected and placed in beakers, with 30 zebrafish in each beaker (experimental group). FEAK was given in aqueous solution (concentrations as shown in Table 1 and Table 2), and 20.0 μg/mL of pioglitazone hydrochloride was used as a positive control. The normal control group and model control group were set up at the same time. After establishing the zebrafish hyperglycemia model and treating the zebrafish with FEAK at 28 °C for 48 h, the zebrafish were washed three times with fish culture water, and the glucose level of zebrafish was detected using a blood glucose meter. The auxiliary hypoglycemic efficacy was evaluated by statistical analysis of the results obtained using this indicator.

### 3.6. Evaluation of Hypolipidemic Effect of FEAK in C. elegans Model

First, *Escherichia coli* (*E. coli)* solution with FEAK was prepared as follows: 500.0 mg/mL mother liquor of FEAK was prepared with DMSO and stored at −20 °C; 10 μL of mother liquor was added to 10 mL concentrated *E. coli* solution and mixed, then the mixture was added to the nematode growth medium (NGM) agar plate and dried by airing for later use. The DMSO control group received 10 μL DMSO instead of FEAK.

The effect of FEAK on lipid deposition was observed by determining the green fluorescence of the dhs-3::gfp mutants. The animals were first synchronized at the L1 stage and then divided into groups. About 50 nematodes were cultured in each plate in a constant temperature incubator set to 20 °C for 6 days. The animals were then washed with M9 buffer, anesthetized with 40 mmol/L imidazole, observed, and photographed under fluorescence microscope. The average fluorescence intensity was calculated using ImageJ.

### 3.7. Statistical Analysis

Each experimental group was set up in three parallel groups. Statistical results were expressed as mean ± SE. SPSS 26.0 software (IBM, 2022, Shanghai, China). was used for statistical analysis, with *p* < 0.05 indicating a statistically significant difference. GraphPad Prism 9.0 software (GraphPad Software, 2021, Shanghai, China) was used for plotting.

## 4. Conclusions

This study aimed to investigate the effect of FEAK on regulating glucose and lipid metabolism. The hypoglycemic and hypolipidemic activities of FEAK were analyzed through a series of in vitro and in vivo experiments, including assay testing the inhibition of α-amylase and cholesterol esterase, the determination of the intracellular TC and TG levels and glucose uptake in HepG2 cells, and the evaluation of hypoglycemic and hypolipidemic efficacy in vivo using zebrafish and *C. elegans.* We found that FEAK is rich in flavonoids, including aureusidin, xanthoangelol, kaempferol, luteolin, and quercetin. We also found that 40 mg/mL of the FEAK showed an inhibition rate of 57.13 ± 1.88% against α-amylase and that 15 mg/mL of the FEAK showed a maximum inhibition rate of 72.11 ± 1.69% against cholesterol esterase. Further, 150 μg/mL of FEAK decreased intracellular TC levels in the HepG2 cells by 33.86% (*p* < 0.001), and 150 μg/mL of the FEAK decreased the intracellular TG levels in the HepG2 cells by 27.89% (*p* < 0.001). When the concentration of the FEAK reached 100 mg/mL, the contrast value indicating the glucose uptake reached its highest value of 68.12% (*p* < 0.01). Moreover, 500 μg/mL of FEAK decreased the blood glucose levels of zebrafish by 57.7% (*p* < 0.001), similar to the positive control drug pioglitazone hydrochloride. Finally, 500 μg/mL of the FEAK decreased the fluorescent intensity of *C. elegans* by 17% (*p* < 0.001) compared to that of the DMSO group. These findings provide strong evidence that FEAK has hypoglycemic and hypolipidemic activity and could be a promising natural product with potential value for the development of functional foods or drugs to prevent or treat type 2 diabetic or hyperlipidemia. However, the mechanism of the hypoglycemic and hypolipidemic effects of FEAK is not very clear, and further research is needed to understand the detailed action mechanism.

## Figures and Tables

**Table 1:** Component analysis of flavonoids in the ethanol extract of *Angelica keiskei*.

Compounds	Class	Relative Quantification (%) (Mean ± SE)
Aureusidin-4-*O*-glucoside	Aurones	5.395 ± 0.082
Xanthoangelol	Chalcones	3.995 ± 0.041
4-Hydroxyderricin	Chalcones	3.767 ± 0.015
Kaempferol-3-*O*-(6″-malonyl)glucoside	Flavonols	3.622 ± 0.116
Kaempferol-3-*O*-(6″-malonyl)galactoside	Flavonols	3.482 ± 0.065
Luteolin-7-*O*-rutinoside	Flavones	3.311 ± 0.212
Kaempferol-3-*O*-glucoside-7-*O*-rhamnoside	Flavonols	3.251 ± 0.007
Luteolin-7-*O*-neohesperidoside (Lonicerin)	Flavones	3.172 ± 0.003
Kaempferol-3-*O*-neohesperidoside	Flavonols	3.170 ± 0.072
Kaempferol-3-*O*-glucorhamnoside	Flavonols	3.125 ± 0.058
Diosmetin-7-*O*-galactoside	Flavones	2.888 ± 0.034
Quercetin-5-*O*-β-d-glucoside	Flavonols	2.805 ± 0.095
6-*C*-MethylKaempferol-3-glucoside	Flavones	2.755 ± 0.009
Diosmetin-7-*O*-glucoside	Flavones	2.750 ± 0.028
Luteolin-7-*O*-glucoside (Cynaroside)	Flavones	2.739 ± 0.406
Hispidulin-7-*O*-Glucoside	Flavones	2.678 ± 0.027
Chrysoeriol-7-*O*-(6″-malonyl)glucoside	Flavones	1.997 ± 0.326
Diosmetin-7-*O*-rutinoside (Diosmin)	Flavones	1.995 ± 0.015
Luteolin-4′-*O*-glucoside	Flavones	1.851 ± 0.171
Quercetin-3-*O*-galactoside (Hyperin)	Flavonols	1.785 ± 0.062
Cyanidin-3-*O*-glucoside (Kuromanin)	Anthocyanidins	1.688 ± 0.191
Quercetin-3-*O*-glucoside (Isoquercitrin)	Flavonols	1.683 ± 0.038
Hispidulin-7-*O*-(6″-*O*-*p*-Coumaroyl)Glucoside	Flavones	1.650 ± 0.055
Kaempferol-3-*O*-glucoside (Astragalin)	Flavonols	1.644 ± 0.036
Dihydrokaempferide	Flavanonols	1.527 ± 0.080
Luteolin-3′-*O*-glucoside	Flavones	1.506 ± 0.018
Luteolin-7,3′-di-*O*-glucoside	Flavones	1.394 ± 0.053
Isobavachalcone	Chalcones	1.36 ± 0.028
Kaempferol-3-*O*-galactoside-4′-*O*-glucoside	Flavonols	1.297 ± 0.041
Xanthoangelol F	Chalcones	1.277 ± 0.044
Yuanhuanin	Flavones	1.216 ± 0.147
Quercetin-7-*O*-glucoside	Flavonols	1.172 ± 0.014
Quercetin-4′-*O*-glucoside (Spiraeoside)	Flavonols	1.165 ± 0.027
Hesperetin-5-*O*-glucoside	Flavanones	1.145 ± 0.028

**Table 2:** Results of cell fluorescence intensity.

Sample	Fluorescence Intensity	δ (Contrast Value)
0 mg/mL	0.1208 ± 0.0211 ^a^	
10 mg/mL	0.1237 ± 0.0272 ^a^	2.40%
25 mg/mL	0.1573 ± 0.0108 ^b^	26.66%
50 mg/mL	0.1711 ± 0.0082 ^b^	41.64%
100 mg/mL	0.2031 ± 0.0008 ^c^	68.12%

The sample without FEAK was used as the control group used to calculate the δ value according to the Equation (2) shown in the Section of Materials and Methods. Note: the different lowercase letters in the fluorescence intensity test group represent the existence of significant differences at *p* < 0.05 in the different concentration group of the FEAK.

**Table 3:** Evaluation of MTC (*n* = 30).

Group	Concentration (μg/mL)	Mortality (%)	Phenotype
Normal control group	-	0	No obvious abnormality
Model control group	-	0	No obvious abnormality
FEAK group	31.2	0	The state is similar to that of the model control group
	62.5	0	
	125	0	
	250	0	
	500	0	

**Table 4:** Experimental result of FEAK-assisted hypoglycemic effect (*n* = 10).

Group	Concentration (μg/mL)	Blood Glucose Value (mmol/L, Mean ± SE)
Normal control group	-	0.92 ± 0.04 ***
Model control group	-	2.31 ± 0.13
Positive control group	20	1.07 ± 0.04 ***
Sample group	125	2.00 ± 0.13
	250	1.65 ± 0.10 *
	500	1.31 ± 0.05 ***

Note: the normal control group was prepared with pure water; the model control group was prepared with 0.15% yolk powder solution in the daytime, and 3% glucose solution was added in the evening after the removal of the yolk powder solution; the positive control group was prepared with pioglitazone hydrochloride tablets based on the model control group; and the sample groups were prepared with different concentrations of FEAK based on the model control group. Statistical differences between groups were obtained by comparing the data of each group with the model control group, * *p* < 0.05, *** *p* < 0.001.

**Table 5:** Inhibition system of α-amylase activity.

Tube	α-Amylase Solution (μL)	Sample (μL)	Starch Solution (μL)	PBS (μL)
Blank 1	300	-	300	150
Blank control 2	-	-	300	450
Sample 3	300	150	300	-
Sample control 4	-	150	300	300

**Table 6:** The amount of additive agents for cholesterol esterase inhibitory activity measurement.

Tube	Cholesterol Esterase Solution (μL)	Sample (μL)	PNPB (μL)	Buffer (mL)
Blank 1	50	-	10	1
Blank control 2	-	-	10	1
Sample 3	50	25	10	1
Sample control 4	-	25	10	1

## Data Availability

Not applicable.

## Sample Availability

Samples of the plant material are available from the authors.
